# PTK6 inhibition promotes apoptosis of Lapatinib-resistant Her2^+^ breast cancer cells by inducing Bim

**DOI:** 10.1186/s13058-015-0594-z

**Published:** 2015-06-19

**Authors:** Sun Hee Park, Koichi Ito, William Olcott, Igor Katsyv, Gwyneth Halstead-Nussloch, Hanna Y. Irie

**Affiliations:** Division of Hematology and Medical Oncology, Department of Medicine, Tisch Cancer Institute, Icahn School of Medicine at Mount Sinai, 1468 Madison Avenue, New York, NY USA; Department of Oncological Sciences, Tisch Cancer Institute, Icahn School of Medicine at Mount Sinai, 1468 Madison Avenue, New York, NY USA

## Abstract

**Introduction:**

Protein tyrosine kinase 6 (PTK6) is a non-receptor tyrosine kinase that is highly expressed in Human Epidermal Growth Factor 2^+^ (Her2^+^) breast cancers. Overexpression of PTK6 enhances anchorage-independent survival, proliferation, and migration of breast cancer cells. We hypothesized that PTK6 inhibition is an effective strategy to inhibit growth and survival of Her2^+^ breast cancer cells, including those that are relatively resistant to Lapatinib, a targeted therapy for Her2^+^ breast cancer, either intrinsically or acquired after continuous drug exposure.

**Methods:**

To determine the effects of PTK6 inhibition on Lapatinib-resistant Her2^+^ breast cancer cell lines (UACC893R1 and MDA-MB-453), we used short hairpin ribonucleic acid (shRNA) vectors to downregulate PTK6 expression. We determined the effects of PTK6 downregulation on growth and survival in vitro and in vivo, as well as the mechanisms responsible for these effects.

**Results:**

Lapatinib treatment of “sensitive” Her2^+^ cells induces apoptotic cell death and enhances transcript and protein levels of Bim, a pro-apoptotic Bcl2 family member. In contrast, treatment of relatively “resistant” Her2^+^ cells fails to induce Bim or enhance levels of cleaved, poly-ADP ribose polymerase (PARP). Downregulation of PTK6 expression in these “resistant” cells enhances Bim expression, resulting in apoptotic cell death. PTK6 downregulation impairs growth of these cells in in vitro 3-D Matrigel^TM^ cultures, and also inhibits growth of Her2^+^ primary tumor xenografts. Bim expression is critical for apoptosis induced by PTK6 downregulation, as co-expression of Bim shRNA rescued these cells from PTK6 shRNA-induced death. The regulation of Bim by PTK6 is not via changes in Erk/MAPK or Akt signaling, two pathways known to regulate Bim expression. Rather, PTK6 downregulation activates p38, and pharmacological inhibition of p38 activity prevents PTK6 shRNA-induced Bim expression and partially rescues cells from apoptosis.

**Conclusions:**

PTK6 downregulation induces apoptosis of Lapatinib-resistant Her2^+^ breast cancer cells by enhancing Bim expression via p38 activation. As Bim expression is a critical biomarker for response to many targeted therapies, PTK6 inhibition may offer a therapeutic approach to treating patients with Her2 targeted therapy-resistant breast cancers.

**Electronic supplementary material:**

The online version of this article (doi:10.1186/s13058-015-0594-z) contains supplementary material, which is available to authorized users.

## Introduction

Patients with breast cancers of specific subtypes are at higher risk for recurrence. Human epidermal growth factor receptor 2 (Her2)^+^ breast cancer is a higher risk subtype that constitutes 20–30 % of all breast tumors. Targeted therapies such as Herceptin and Lapatinib have improved recurrence-free survival and helped control metastatic or recurrent disease (as reviewed [1]). However, response to these therapies is not uniform and resistance, either intrinsic or acquired, remains a significant clinical challenge. Strategies to treat breast cancers that are no longer sensitive to these targeted therapies could translate into improved outcomes for patients.

We initially identified protein tyrosine kinase 6 (PTK6) as a critical mediator of anoikis resistance of breast cancer cells in a functional genomic screen designed to identify regulators of anchorage-independent survival [2]. PTK6, a member of a distinct family of non-receptor tyrosine kinases distantly related to Src kinases, is expressed in breast cancers and multiple other cancer types [3–7]. We reported that PTK6 transcript expression has prognostic significance; higher levels of PTK6 are associated with adverse outcomes independently of other factors such as nodal status. Among the molecular subtypes of breast cancer, estrogen receptor (ER)^+^ and Her2^+^ cancers express the highest levels of PTK6 transcript [2].

PTK6 is a non-receptor tyrosine kinase composed of an amino-terminal SH3 domain, SH2 domain, and carboxyl-terminal kinase domain (as reviewed [6, 7]). PTK6 promotes oncogenic phenotypes including enhanced proliferation, enhanced anoikis resistance, regulation of autophagy, epithelial-mesenchymal transition, and migration/invasion, via kinase activity-dependent and possibly independent mechanisms [2, 6–11]. There are increasing numbers of PTK6 kinase substrates, including Sam68, Stat3/5b, BKS, Fak, Cbl, and paxillin, many of which are known to play critical roles in oncogenic signaling [12–19]. Unlike the distantly related src kinases, PTK6 lacks a myristylation sequence. Therefore, PTK6 exhibits a broader range of cellular localization that could impact its activities; PTK6 protein has been detected in the nucleus, cytosol, and membranes of cells [4, 10, 20]. The preferential localization pattern of PTK6 appears to differ between normal vs tumor cells, which could account for differential access to substrates and differential activities in these contexts; while PTK6 is expressed in the nucleus of normal luminal prostate epithelial cells, PTK6 is largely cytosolic in more aggressive prostate cancer cells [4, 12].

PTK6 impacts survival of both normal and cancer cells, and may seemingly play contradictory roles in these two contexts. In normal intestinal epithelial cells, PTK6 is required for apoptosis induced by DNA damage following UV irradiation [21]. In contrast, in many tumor model systems PTK6 promotes survival. For example, enhanced PTK6 expression inhibits anoikis and autophagic death following matrix detachment and promotes soft agar colony growth [2, 9, 17, 22]. Furthermore, downregulation of PTK6 enhances anoikis of breast, ovarian and prostate cancer cells [2, 17]. PTK6 may also regulate sensitivity to targeted therapeutics. In the studies of Xiang et al., overexpression of PTK6 in ErbB2^+^ MCF-10A cells suppressed the growth inhibitory effects of Lapatinib treatment [23]. However, the precise molecular mechanisms by which PTK6 regulates survival and specifically the apoptotic machinery, of Her2-targeted therapy-resistant cells have not yet been elucidated.

In this study, we sought to determine the effects of PTK6 inhibition on growth and survival of Lapatinib-resistant Her2^+^ breast cancer cells. We demonstrate that PTK6 downregulation induces apoptosis of these cells by enhancing Bim protein expression. Induction of Bim is critical, as downregulation of Bim expression prevents PTK6 shRNA-induced apoptosis. We also present evidence for p38 activation as a mechanism for PTK6 shRNA-induced Bim induction, and provide the first link between PTK6 and the intrinsic apoptotic pathway.

## Methods

### Antibodies and reagents

GAPDH, cleaved PARP, phospho-ERK1/2, phospho-AKT (Ser473), phospho-p38 (Thr180/Tyr182), phospho-hsp27 (Ser82), phospho-JNK (Thr183/Tyr185), p-c-Jun (Ser73), p-ATF2 (Thr71), α-tubulin, phospho-Her2 (Tyr1289), phospho-Her2 (Tyr877), Hsp70, Bcl-2, Bcl-xL, Mcl-1, pro-apoptotic Bcl-2 family members (Puma, phospho-Bad (Ser112), Bid), and total p38 antibodies were purchased from Cell Signaling (Danvers, MA, USA). PTK6 (D7), PTK6 (C18), anti-rabbit-hrp, anti-mouse-hrp antibodies, and Protein A/G Plus-agarose (sc-2003) were purchased from Santa Cruz Biotechnology, Inc (Dallas, TX, USA). Bim antibody was purchased from Abcam (Cambridge, MA, USA). Growth factor-reduced Matrigel^TM^ and Z-VAD-FMK were purchased from BD Bioscience (Franklin Lakes, NJ, USA). Lipofectamine 2000 and Plus reagent were purchased from Life Technologies (Grand Island, NY, USA). Lapatinib, SB203580, and SP600125 were purchased from Sellekchem (Houston, TX, USA).

### RNAi

PTK6 Mission shRNAs (TRCN0000021549 (49), TRCN0000021552 (C9), TRCN0000196912 (12), TRCN0000199853 (53)) and Bim shRNAs (TRCN0000001051 (51), TRCN0000001054 (54)) were purchased from Sigma Aldrich (St. Louis, MO, USA).

### Cell lines

MDA-MB-453, UACC893, SKBR3, and HCC1954 were purchased form ATCC (Manassas, VA, USA). MDA-MB-453 and UACC893 cells were maintained in complete DMEM medium supplemented with 10 % fetal bovine serum and penicillin/streptomycin. The Lapatinib-resistant cell line, UACC893R1, was generated by culturing parental UACC893 cells continuously over 6 months in the presence of increasing concentrations of Lapatinib (up to 5 μM). These cells were then maintained in DMEM complete medium in the presence of 1 μM Lapatinib. SKBR3 and HCC1954 cells were maintained in complete McCoy’s 5A and RPMI medium, respectively, supplemented with 10 % fetal bovine serum and penicillin/streptomycin.

### Viral infections

Lentivirus was generated by co-transfecting 293T cells with lentiviral vector, Δ8.9, and pCMV-VSV-G using Lipofectamine 2000 and Plus reagent as described in Irie et al. [2]. Supernatants were collected and frozen at −80 °C overnight. Retrovirus was generated by transfecting 100-mm plates of 293-GPG cells with retroviral vectors using Lipofectamine 2000 according to established protocols [24]. Virus was collected, filtered and stored at −80 °C. Cells were infected with virus by spin-infection at 2,250 rpm for 30 minutes at room temperature followed by overnight incubation at 37 °C. Infected cells were selected in the presence of antibiotics (puromycin or G418) purchased from InvivoGen (San Diego, CA, USA).

### Quantitative real-time PCR

GAPDH and β-Actin primers were purchased from Qiagen (Venlo, Netherlands). β-2M (Hs00984230_m1), PTK6 (Hs00963386_m1), and Bim (Hs00708019_s1) gene expression assays were purchased from Applied Biosystems (Grand Island, NY, USA). The Bim gene expression assay detects all isoforms of Bim transcript (Bim_EL_, Bim_L_, and Bim_S_). For real-time PCR, RNA was extracted from cell lines using RNeasy kit (Qiagen) and cDNA was synthesized using Taqman cDNA synthesis kit and oligo-dt (16) primers (Life Technologies). Taqman PCR reactions were run using 2 × Taqman universal master mix II (Applied Biosystems, Cat. number 4440040), 5 μl of undiluted cDNA, and 1 μl of PTK6 gene expression assay (protocol used: UNG incubation (50 °C, 2 minutes), polymerase activation (95 °C, 10 minutes), 40 cycles of denaturation (95 °C, 15 sec) and annealing/extension (60 °C, 1 minute)). For other genes, the reactions were performed using 2 × Power SYBR green PCR master mix (Life Technologies), 5 μl of two-fold diluted cDNA, and 2.5 μl of 10 μM primer mix.

### Three-dimensional (3-D) cell growth assays

Eight-well chamber slides (BD Biosciences) were coated with 50 μl of growth factor-reduced Matrigel^TM^; 400 μl of complete growth media containing 4,000 cells were added to each well coated with Matrigel^TM^. All samples were set up in triplicates. The chamber slides were incubated at 37 °C and re-fed every 3–4 days with complete growth media. Cells were imaged using the Axiovert 25 inverted microscope (Carl Zeiss AB).

### Western blots

Protein lysates were prepared using 1 % NP40 Lysis Buffer (Boston Bioproducts, Ashland, MA, USA) and quantified by bicinchoninic acid (BCA) assay. Lysates were resolved using 4–12 % Bis-Tris gradient gels (Life Technologies), transferred at 100 V onto polyvinylidene fluoride (PVDF) membranes using transfer buffer solution (Boston Bioproducts) containing 10 % methanol. The membranes were blocked in 5 % BSA/TBS + 0.05 % Tween solution at room temperature and incubated in primary antibody overnight at 4 °C. Membranes were incubated with secondary antibody (1:2,000) for 1 h at room temperature and were washed in 1 × TBS+ 0.05 % Tween. Blots were developed using ECL (Pierce).

### Growth curve analysis

We plated 5 × 10^4^ MDA-MB-453 or UACC893R1 cells infected with either control or PTK6 shRNAs onto 12-well plates in triplicate. The number of live cells was counted every 3–4 days to generate the growth curves. Experiments were performed two or three times with UACC893R1 and MDA-MB-453 cells, respectively. For growth curve analysis in the presence of Lapatinib, 1 × 10^5^ UACC893 and UACC893R cells were plated in triplicate in 24-well plates. Cells were treated with either DMSO or Lapatinib (0.5–5 μM). The number of live cells was counted to generate the growth curves.

### Fluorescence-activated cell sorting (FACS)

We detached 5 × 10^5^ cells from the plates using 3mM EDTA/PBS and resuspended in 500 μl of ice-cold PBS. Annexin V-fluorescein isothiocyanate (FITC) staining was performed according to the manufacturer’s protocol (BD Pharmingen, San Diego, CA, USA). For cell cycle profile analysis, cells were fixed using 80 % ethanol, stored overnight at 4 °C, and then spun at 1,500 rpm for 5 minutes at 4 °C using a centrifuge (Eppendorf 5810R). Pellets were washed with cold PBS + 1 % serum, mixed, spun for 5 minutes at 1,200 rpm, and stained with propium iodide (PI)/RNase solution (BD Pharmingen). All analysis was performed using FACS Diva software on a BD FACSCanto II flow cytometer.

### Soft agar assays

Base agar was prepared with 0.8 % agarose (Lonza, Basel, Switzerland) at 42 °C. Equal volumes of agarose and 2 × complete growth media were mixed and plated. Top agar (final concentration of 0.4 %) was prepared using a 1:1 mixture of 0.8 % agarose and 2 × growth medium at 37 °C. Cells resuspended in top agar were plated and cultured for 30 days. Cells were re-fed one to two times a week with fresh media.

### Tumor xenografts

We resuspended 5 × 10^5^ UACC398R1 cells infected with either control or PTK6 shRNA lentivirus in 100 μl of growth-factor-reduced Matrigel^TM^ on ice. Cell suspensions were injected subcutaneously into the flanks of 6-week-old female nude (nu/nu) mice (Charles River Laboratories). Tumor measurements were performed twice weekly and tumor volume was calculated using the formula: V =1/2 (L × W^2^). All procedures and studies with mice were performed in accordance with protocols pre-approved by the Institutional Animal Care and Use Committee of Mount Sinai.

### Immunoprecipitation (IP)

We lysed 5 × 10^5^ UACC893R1 cells infected with either control or PTK6 shRNA lentivirus in IP lysis buffer (NP-40 lysis buffer (Boston Bioproducts: BP-431), PMSF, leupeptin, aprotinin, NaF, Na_3_VO_4,_ and phosSTOP (Roche, Basel, Switzerland)) and incubated at 4 °C for 20 minutes. Cell extract was prepared by spinning at full speed for 20 minutes at 4 °C using a centrifuge (Eppendorf Centrifuge 5424R). Supernatant was pre-cleared with 50 % slurry beads (pre-equilibrated with IP lysis buffer) for 30 minutes, incubated with PTK6 antibody (C18) for 2 h, and incubated with 35 μl of 50 % slurry beads for 1 h in a rotating wheel at 4 °C. Beads were washed three times with IP wash buffer (IP lysis buffer without protease inhibitor) and boiled with 2 × SDS sample buffer for 3 minutes at 95 °C. The samples were analyzed by western blotting following the above protocol.

### Consent statement

We confirm that this study did not involve human patients and no consent was necessary.

## Results

### Bim expression is not induced in Lapatinib-resistant Her2^+^ breast cancer cells

**Fig. 1:**
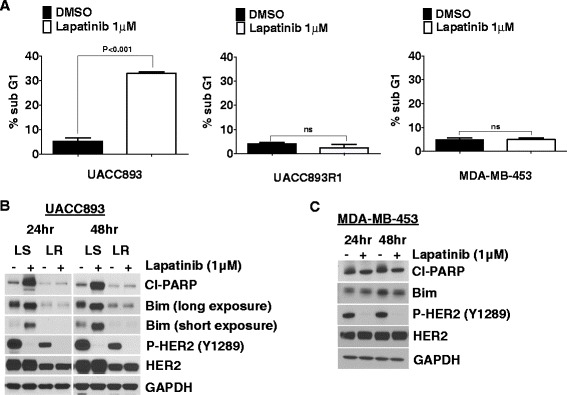
Apoptosis and Bim are not induced by Lapatinib treatment of tyrosine kinase inhibitor (TKI)-resistant human epidermal growth factor receptor 2 (*Her2*)^+^ breast cancer cells. **a** UACC893, UACC893R1 (Lapatinib-resistant variant of UACC893), and MDA-MB-453 cells were grown in monolayer cultures, treated with either dimethyl sulfoxide (*DMSO*) or Lapatinib (1 μM) for 24 h and stained with propidium iodide. Percentage of cells in the sub-G1 population is plotted. **b** Parental UACC893 (indicated as *LS*) or Lapatinib-resistant UACC893R1 cells (indicated as *LR*) were treated with DMSO or Lapatinib (1 μM) for 24 h or 48 h and lysed. Lysates were probed with the indicated antibodies. **c** MDA-MB-453 cells in monolayer cultures were treated with DMSO or Lapatinib (1 μM) for 24 h or 48 h, lysed and probed with the indicated antibodies. Experiments were performed three times and representative data are shown. *ns* not statistically significant

We assessed a panel of Her2^+^ breast cancer cells with respect to their relative sensitivities to treatment with Lapatinib, a small molecule inhibitor of Her2 and other epidermal growth factor receptor (EGFR) family members that is used in clinical practice. Cell death following 24 h of Lapatinib treatment was initially quantified by plotting the percentage of cells in the sub-G1 fraction after staining with PI. The percentage of cells in the sub-G1 fraction varied from 3 % (least sensitive) to 30 % (most sensitive). Consistent with previous reports, some cell lines were relatively sensitive (e.g., SKBR3, HCC1954), while others were more resistant to Lapatinib treatment at baseline (e.g., MDA-MB-453) [25]. We also generated resistant cell lines (UACC893R1) by culturing sensitive lines such as UACC893 in the presence of escalating doses of Lapatinib over 6–12 months. Resistance to Lapatinib was confirmed (Fig. 1a and Additional file 1: Figure S1A and S1B).

Lapatinib treatment of the sensitive, parental UACC893 cells increased expression levels of pro-apoptotic Bim and increased levels of cleaved poly ADP ribose polymerase (PARP), a marker of apoptosis. Levels of PUMA, another pro-apoptotic Bcl2 family member implicated in Lapatinib-induced apoptosis were decreased (Additional file 1: Figure S1C and [26]). Induction of Bim and cleaved PARP was also observed with Lapatinib treatment of other sensitive Her2^+^ breast cancer cell lines, SKBR3 and HCC1954 (Additional file 1: Figure S1D). Interestingly, the level of basal Bim expression was decreased in UACC893R1 cells, the resistant variant of UACC893, when compared to the parental Lapatinib-sensitive UACC893 cells. In contrast to Lapatinib treatment of parental UACC893 cells, treatment of UACC893R1 did not increase expression of Bim above basal levels. Lapatinib treatment of UACC893R1 cells also did not result in enhanced levels of cleaved PARP (above basal) even though Her2 phosphorylation was still effectively inhibited (Fig. 1b). In addition, in MDA-MB-453 cells, which are intrinsically resistant to Lapatinib treatment, Lapatinib did not enhance levels of Bim or apoptosis, as assessed by cleaved PARP detection (Fig. 1c). Therefore, in some Her2^+^ breast cancer cells, resistance may be linked to the inability of Lapatinib treatment to induce Bim and apoptosis, and strategies that enhance Bim expression could induce death of these resistant cells.

### Downregulation of PTK6 inhibits growth and induces death of Lapatinib-resistant Her2^+^ breast cancer cells

We previously reported that PTK6 transcript is highly expressed in the Her2^+^ subtype and downregulation enhances anoikis of Her2^+^ breast cancer cells [2]. Interestingly, in The Cancer Genome Atlas (TCGA)-Breast expression dataset PTK6 expression correlates with genes that negatively regulate programmed cell death (NIH DAVID fold enrichment = 1.85, nominal *p* value = 4.27 × 10^−4^) [27] (Additional file 2: Figure S2). These data led us to hypothesize that PTK6 inhibition induces death of Her2^+^ breast cancer cells, including those that are Lapatinib-resistant, by regulating the expression of apoptosis-related genes.

**Fig. 2:**
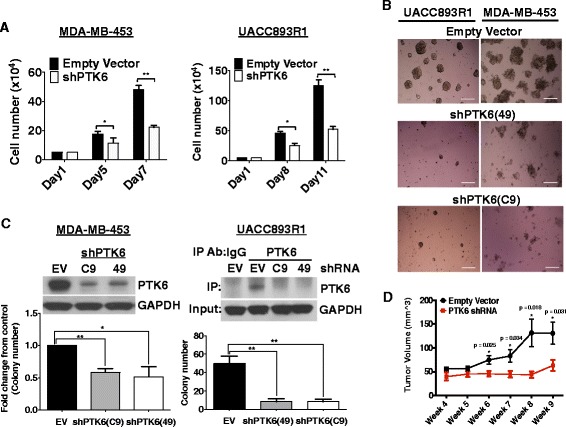
Protein tyrosine kinase 6 (*PTK6*) downregulation inhibits growth of Lapatinib-resistant human epidermal growth factor receptor 2 (Her2)^+^ breast cancer cells. **a** UACC893R1 or MDA-MB-453 cells grown in monolayer cultures expressing control or PTK6 shRNA (C9), were counted at the indicated number of days. **b** Cells expressing either control or two different PTK6 shRNAs (C9, 49) were grown in 3-D Matrigel^TM^ cultures. Cells were re-fed with fresh media every 3–4 days. The figure represents day-16 cultures (UACC893R1) or day-23 cultures (MDA-MB-453). Scale bar 30 μm. **c** Cells expressing control or PTK6 shRNA (C9, 49) were cultured in soft agar assays for 30 days. Colonies greater than 50 square pixels were counted and plotted. Downregulation of PTK6 was confirmed by western analyses for MDA-MB-453 and UACC893R1 cells. **d** UACC893R1 cells expressing control or PTK6 shRNA (C9) were injected subcutaneously into flanks of 6-week-old female nude mice (n = 5/group). Tumor size was measured and recorded every 3–4 days. All figures are representative of three independent experiments except for the xenograft study, which was performed twice; *p <0.05; **p <0.005

**Fig. 3:**
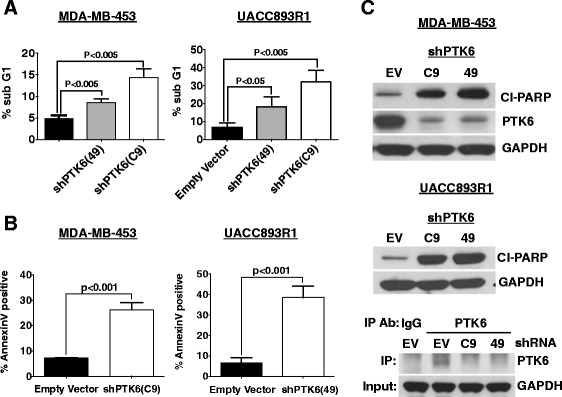
Protein tyrosine kinase 6 (*PTK6*) downregulation induces apoptosis of Lapatinib-resistant human epidermal growth factor receptor 2 (Her2)^+^ breast cancer cells. **a** MDA-MB-453 and UACC893R1 cells expressing control or two different PTK6 shRNA (C9 and 49) were fixed, stained with propium iodide (PI), and analyzed by flow cytometry. The percentage of cells in the sub-G1 population is plotted. **b** MDA-MB-453 and UACC893R1 expressing control or PTK6 shRNA (C9 or 49, respectively for the two cell lines) were stained with Annexin-V and PI, and analyzed by fluorescence-activated cell sorting. **c** Cells expressing control or two different PTK6 shRNAs (C9, 49) were lysed 96 h (MDA-MB-453) or 72 h (UACC893R1) following infection with shRNA lentivirus. Lysates were probed with indicated antibodies. Immunoprecipitation and western blot analysis confirms PTK6 downregulation in UACC893R1 cells. Figures are representative of three independent experiments

We determined the effects of downregulating PTK6 expression on the growth and survival of MDA-MB-453 or UACC893R1 cells. ShRNA-vector-mediated downregulation of PTK6 alone significantly inhibited growth in 2-D monolayer and 3-D Matrigel cultures^TM^ (Fig. 2a, b). PTK6 shRNA expression impaired soft agar colony formation of MDA-MB-453 and UACC893R1 cells (Fig. 2c). PTK6 downregulation also suppressed growth of UACC893R1 primary tumor xenografts (Fig. 2d). The compromised growth of these Lapatinib-resistant Her2^+^ cells in 2-D and 3-D cultures and in vivo is in part due to increased apoptosis; PTK6 shRNA expression enhanced the percentage of cells in the sub-G1 fraction, and Annexin-V-positive cells (Fig. 3a, b and Additional file 3: Figure S3A). In addition, PTK6 downregulation enhanced levels of cleaved PARP (Fig. 3c and Additional file 3: Figure S3B). These data support apoptosis induction as a mechanism by which PTK6 downregulation impairs survival and growth of Lapatinib-resistant Her2^+^ breast cancer cells.

### PTK6 downregulation induces Bim expression, which is required for apoptosis

**Fig. 4:**
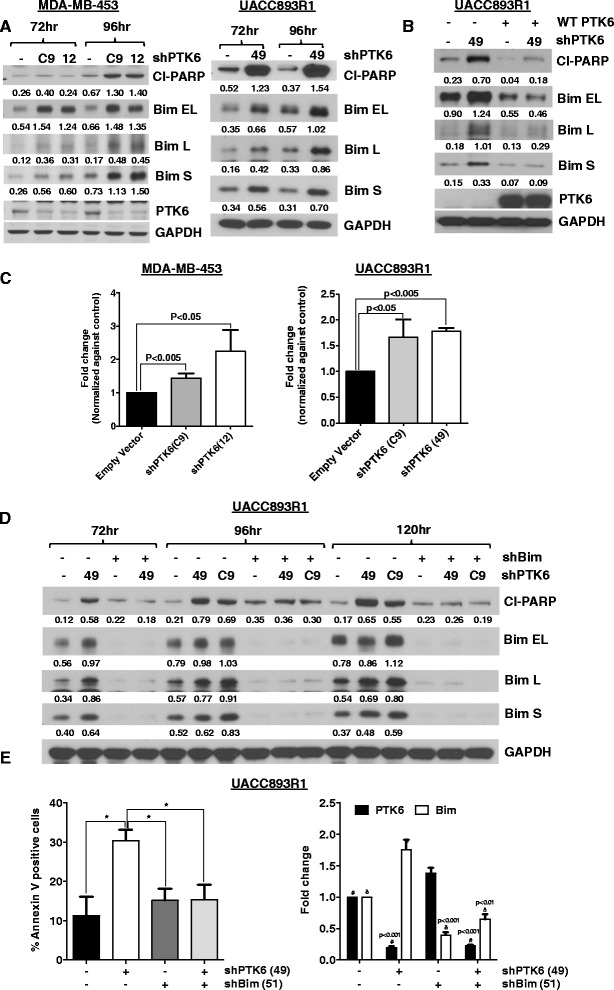
Induction of Bim expression is required for apoptosis following protein tyrosine kinase 6 (*PTK6*) downregulation. **a** MDA-MB-453 and UACC893R1 cells expressing control or PTK6 shRNA(s) (C9, 12 or 49) were lysed at two time points following shRNA lentiviral infection. Lysates were probed with indicated antibodies. **b** UACC893R1 cells expressing either empty or wild-type (WT) PTK6 cDNA vector were super-infected with control or PTK6 shRNA lentivirus that targets the 3′UTR of PTK6 (49). Cells were lysed 96 h after infection and lysates were probed with the indicated antibodies. **c** RNA from UACC893R1 or MDA-MB-453 expressing PTK6 shRNA (C9, 12, or 49) was extracted and levels of Bim transcript were assessed. **d** UACC893R1 cells expressing control or Bim shRNA (51) were superinfected with control or PTK6 shRNA lentivirus. Cells were harvested and lysates were probed with antibodies to cleaved poly ADP ribose polymerase (PARP) and Bim. All experiments were performed three times. Numbers below blots indicate quantification of band intensity performed using Image J, normalized to respective loading control bands. **e** UACC893R1 cells co-infected with the indicated viruses (*EV*, empty vector; PTK6 shRNA 49; Bim shRNA 51) were harvested 96 h after infection, stained with Annexin-V and propium iodide, and analyzed by flow cytometry. The percentage of Annexin-V positive cells is plotted. *Right*, the levels of Bim or PTK6 transcript were evaluated in parallel samples. Statistics were applied to results obtained with triplicate experiments; *P <0.05. P-values were determined by comparing control shRNA to PTK6 and/or Bim shRNA-treated samples: ^δ^PTK6; ^#^Bim

We sought to determine the mechanisms by which PTK6 regulates survival of Lapatinib- resistant Her2^+^ breast cancer cells. As a first step, we evaluated the effect of PTK6 downregulation on expression of pro-and anti-apoptotic members of the Bcl2 family. We did not observe any changes in expression of Mcl-1, Bcl-xL, Bcl-2, Puma, Bid, or phospho-Bad (Additional file 4: Figure S4). However, with PTK6 shRNA expression, we observed an increase in expression of the three major isoforms of Bim, a pro-apoptotic BH3 domain-only member of the Bcl2 family (Bim_EL_, Bim_L_, and Bim_S_); changes were most pronounced for Bim_EL_ and Bim_S_, and to lesser degree for Bim_L_ (Fig. 4a). The enhancement in Bim levels and apoptosis induced by PTK6 shRNA expression can be fully suppressed by co-expression of wild-type PTK6 that cannot be targeted by the PTK6 shRNA vector, supporting the specificity of this regulation (Fig. 4b). Furthermore, Bim induction was observed with PTK6 shRNA expression even in the presence of Z-VAD-FMK, a pan-caspase inhibitor, indicating that the induced Bim expression is not secondary to cell death (Additional file 5: Figure S5). The induction of Bim protein observed with PTK6 shRNA expression is at least in part due to increased transcript levels of Bim, as assessed by quantitative RT-PCR (Fig. 4c).

To address the requirement for Bim induction in apoptosis induced by PTK6 shRNA expression, we co-infected cells with shRNA vectors targeting PTK6 and Bim. Co-infection of Bim shRNA (with either of two independent vectors) rescued PTK6 shRNA-expressing cells from apoptosis, as assessed by levels of cleaved PARP and number of Annexin V-positive cells (Fig. 4d, e, and Additional file 6: Figure S6). These data support a causal, mechanistic link between PTK6 shRNA expression, Bim expression and apoptosis of these Lapatinib-resistant Her2^+^ breast cancer cells.

### PTK6 downregulation enhances Bim expression in part through activation of p38MAPK signaling

**Fig. 5:**
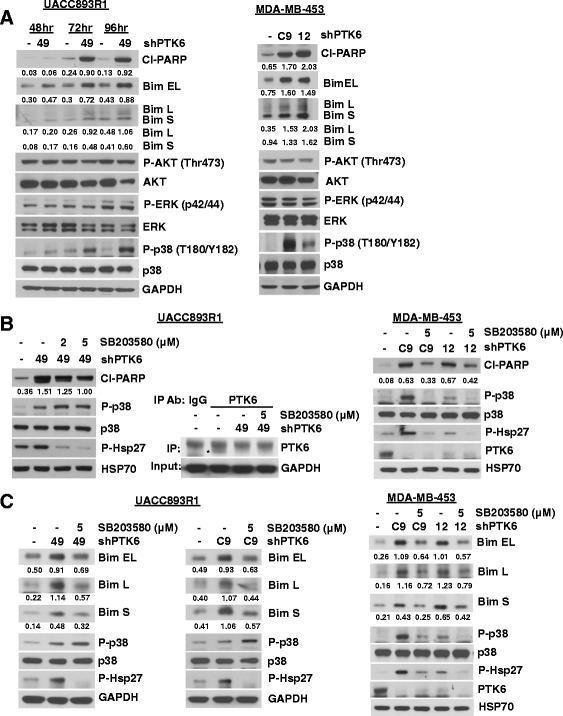
Protein tyrosine kinase 6 (*PTK6*) regulates Bim in part through p38 mitogen-activated protein kinase (*MAPK*) activation. **a** UACC893R1 or MDA-MB-453 cells expressing either control or PTK6 shRNA (49, C9 or 12) were lysed. Lysates were probed with indicated antibodies. **b** UACC893R1 or MDA-MB-453 cells expressing either control or PTK6 shRNA (49, C9 or 12) were cultured in the presence of dimethyl sulfoxide (DMSO) or SB203580 (p38 inhibitor). Cells were lysed for 72 h (UACC893R1) or 96 h (MDA-MB-453) after infection, and lysates were probed with the indicated antibodies. Immunoprecipitation and western blot analysis were performed to show PTK6 downregulation in UACC893R1 cells; *PTK6 that was immunoprecipitated. **c** Cells expressing either control or PTK6 shRNAs (49, C9 or 12) were treated with either DMSO or SB203580. Cells were lysed 72 h (UACC893R1) or 120 h (MDA-MB-453) after shRNA lentiviral infection. Lysates were probed with the indicated antibodies. Experiments were performed three times

To determine the signaling pathways responsible for PTK6 downregulation-mediated Bim induction in UACC893R1 and MDA-MB-453 cells, we examined the status of major signaling pathways activated downstream of Her2 that have been implicated in survival, and Bim regulation [26, 28]. Interestingly, PTK6 downregulation did not consistently affect either Akt or Erk/mitogen-activated protein kinase (MAPK) signaling, two pathways known to regulate Bim expression (Fig. 5a). In contrast, we observed robust activation of p38 MAPK with PTK6 downregulation (Fig. 5a).

To determine if p38 MAPK kinase activation plays a role in Bim induction and apoptosis following PTK6 expression downregulation, UACC893R1 or MDA-MB-453 cells expressing PTK6 shRNA were treated with a pharmacological inhibitor of p38 (SB203580) and effects on Bim expression and apoptosis were assessed. Inhibition of p38 activity by SB203580 was confirmed by assessing the level of phosphorylation of Hsp27, a direct substrate of p38; the inhibition of p38 activity was nearly complete at 5-μM concentration (Fig. 5b). SB203580 treatment partially rescued cells from apoptosis in a dose-dependent manner, as assessed by levels of cleaved PARP (Fig. 5b). In addition, treatment of PTK6 shRNA-expressing UACC893R1 cells with SB203580 prevented PTK6 downregulation-induced expression of all Bim isoforms (Fig. 5c). Similarly in MDA-MB-453 cells, treatment with the p38 inhibitor SB203580 prevented PTK6 shRNA-induced Bim expression and apoptosis (Fig. 5b and c). These effects of p38 inhibitor treatment are not due to generalized inhibition of stress-related kinases. JNK is also activated in response to PTK6 downregulation (Additional file 7: Figure S7A). However, treatment with SP600125, a JNK inhibitor, failed to rescue cells from apoptosis or prevent Bim induction at doses that inhibited anisomycin-induced JNK activity (Additional file 7: Figure S7B and C). Taken together, our results support a role for activation of p38MAPK in Bim expression and apoptosis induced by PTK6 downregulation.

## Discussion

The treatment of breast cancers resistant to current standard therapies poses significant clinical challenges. Cancers intrinsically possess or develop mechanisms to evade the death-inducing effects of cytotoxic agents, as well as targeted therapies. Treatment resistance contributes to the development of recurrent or metastatic breast cancers, which are responsible for the majority of deaths due to breast cancer. Therefore, strategies to effectively inhibit the growth and induce death of breast cancer cells resistant to currently available targeted therapies could lead to novel therapeutic options for patients with breast cancer. In this study, we report for the first time that inhibition of PTK6 induces apoptotic cell death of Her2^+^ breast cancer cells that are relatively resistant to Lapatinib at baseline or after continuous treatment in the presence of this Her2 tyrosine kinase inhibitor (TKI). Apoptosis is induced via enhanced expression of Bim, a BH3-only member of the Bcl2 family via a p38-dependent mechanism.

Our studies show for the first time a link between PTK6, pro-apoptotic Bim and apoptosis of Her2^+^ breast cancer cells. Bim, which is expressed as three major isoforms (Bim_EL_, Bim_L_, and Bim_S_), is a regulator of the mitochondrial (intrinsic) apoptotic pathway ([29] and also reviewed in [30]). Bim is emerging as a biomarker of sensitivity to targeted therapies, including those that target EGFR family members such as Lapatinib. Bim is frequently downregulated in cancers and lower levels of Bim expression are associated with poorer response to targeted therapy treatment [31]. Tumors with relatively lower levels of Bim due to a common deletion polymorphism are also more resistant to EGFR tyrosine kinase inhibitors [32].

Induction of Bim expression tips the functional balance of interacting Bcl2 family members in favor of apoptosis. Bim induction is required for targeted therapy-induced apoptosis of colon, lung, and breast cancers; for example, siRNA-mediated Bim downregulation impaired apoptosis of Her2^+^ BT474 cells in response to Lapatinib treatment [26, 28, 33, 34]. In studies presented in this report, we found that Bim expression is not significantly induced in Her2^+^ breast cancer cells that are resistant to Lapatinib, either at baseline or acquired through continuous exposure to Lapatinib, and this lack of induction correlates with lack of apoptosis in response to Lapatinib treatment. Interestingly, we did not observe induction of PUMA, another BH3 only protein implicated in Lapatinib-induced apoptosis, in either the Lapatinib-sensitive or resistant cell lines evaluated in this report (Additional file 1: Figure S1C and [26]).

Strategies that enhance Bim expression, such as PTK6 inhibition, could therefore be an effective strategy to induce death of TKI-resistant Her2^+^ breast cancer cells. Bim is regulated on multiple levels via transcriptional and post-transcriptional mechanisms. Transcription factors such as Foxo3a, NF-κB, c-Myc, CHOP, and AP-1 are known to regulate Bim transcription [35–39]; these are in turn regulated by major survival signaling molecules, such as Erk/MAPK, Akt, and p38 [39–42]. Bim transcript levels are also influenced by epigenetic modifications of the BIM locus and by microRNA-dependent suppression [43–46]. Post-transcriptionally, the stability of Bim protein is regulated by Erk-dependent ubiquitination and proteasome-dependent degradation [47]. PTK6 inhibition is one approach to induce death by enhancing the expression of Bim protein to levels sufficient to induce apoptosis in Lapatinib-resistant cells. The increased expression of Bim protein is at least partially accounted for by increased levels of Bim transcript in PTK6 shRNA-expressing cells. Future studies will elucidate the specific Bim transcriptional programs regulated by PTK6.

Our studies also show for the first time a link between p38 signaling and PTK6-dependent Bim regulation in Her2^+^ breast cancer cells. Following PTK6 downregulation, we did not consistently observe changes in Erk/MAPK or Akt signaling. Rather, p38 MAPK was robustly activated and contributes to the induction of Bim protein expression and apoptosis. The rescue of cells from apoptosis and inhibition of Bim expression by p38 inhibitor treatment is not due to generalized rescue of stress signaling as inhibition of JNK, which is also activated by PTK6 shRNA expression, does not block apoptosis or Bim induction. The pro-apoptotic roles of p38 in the setting of cellular stress are well documented. P38 induces Bim activity and apoptosis via direct phosphorylation at Serine 65 following Sodium arsenite treatment [48]. In addition, p38 activity led to increased Bim transcription following glucocorticoid treatment of lymphoblastic leukemia cells [49]. However, in other studies, p38 promotes cellular survival, for example, in response to DNA damage or activation of growth factor receptors (e.g., IGF-R1) due to ionizing radiation [50, 51]. Our results are also interesting in light of a previous study showing that PTK6 promotes p38 MAPK activation, subsequent Cyclin D1 expression and migration in the context of heregulin- or EGF-stimulated breast cancer cells [52]. These seemingly conflicting roles of p38 in normal and cancer cell phenotypes have been repeatedly observed. P38 may play a role in pro- or anti-proliferative functions, as well as pro- or anti-apoptotic signaling depending on the cell-type-specific context, the specific stimuli that are used to activate p38, and the intensity or duration of its activation. Future studies are aimed at further dissecting the mechanism by which PTK6 inhibition activates p38 signaling, as well as the mechanisms responsible for p38-mediated regulation of Bim and apoptosis downstream of PTK6.

Recently Ludyga et al. showed that downregulation of PTK6 expression, alone or in combination with Her2 downregulation in Lapatinib and Tratuzumab-resistant, JIMT-1 breast cancer cells inhibited their proliferation without causing cell death [53, 54]. The lack of apoptosis following PTK6 downregulation contrasts with our findings in two independent Her2^+^ cell lines that are resistant to Lapatinib treatment. This may potentially be due to several factors: 1) the relatively high levels of autophagy reported in JIMT-1 cells which may protect cells from apoptosis-inducing stimuli [55]; 2) the differential expression of Bcl2 family members and other proteins (e.g., high MUC4 expression in JIMT-1 cells) that could modify the threshold for apoptosis induction [56]; and 3) differences in the genetic background of these cells (e.g., PTEN status) that could modify apoptotic responses [57, 58]. Nevertheless, it is encouraging that our studies are complementary in demonstrating the efficacy of PTK6 inhibition in inhibiting the growth of Her2 targeted therapy-resistant breast cancer cells, and future studies are aimed at identifying biomarkers associated with cytostatic vs. cytocidal responses to PTK6 downregulation.

The studies presented in our current report support the clinical translation of PTK6 inhibition. There are already several small molecule inhibitors of PTK6 that have been developed and they potently inhibit kinase activity in vitro [59–61]. As these become available, it will be critical to assess whether inhibition of kinase activity phenocopies our results with shRNA expression vectors. The kinase dependency of PTK6-induced oncogenic phenotypes has previously been reported by us and others; in our previous report, we showed that the ability of PTK6 to enhance anoikis resistance when overexpressed in immortalized breast epithelial cells was dependent on PTK6 kinase activity [2]. Kinase activity of overexpressed PTK6 is also responsible for enhanced cell migration and invasion of MDA-MB-231 triple-negative breast cancer cells [19]. These kinase-dependent functions are likely due to phosphorylation and/or activation of an increasing number of PTK6 substrate molecules, including Sam68, Stat3/5b, paxillin, BKS/STAP2, p130CAS, AKT, β-catenin, and p190RhoGAP [12–17, 19, 20, 62–64]. However, Harvey et al. have also reported that overexpression of a kinase-inactive PTK6 is able to enhance proliferation of T47D breast cancer cells relative to vector control-expressing cells [9]. As these studies did not include simultaneous evaluation of a kinase-active PTK6, it is difficult to specifically assess the relative contribution of kinase activity to enhanced proliferation. Nevertheless, it is possible that PTK6 is able to regulate proliferation via protein-protein interactions independently of kinase activity. Small molecule inhibitors of PTK6 should prove useful in determining the role of PTK6 kinase activity in Bim and apoptosis regulation of Her2^+^ breast cancer cells.

## Conclusions

In conclusion, our study supports PTK6 inhibition as a strategy to induce apoptosis of Lapatinib-resistant Her2^+^ breast tumors by enhancing expression of pro-apoptotic Bim that may be suppressed via multiple mechanisms in breast cancers. PTK6 downregulation induces Bim and apoptosis by stimulating p38 MAPK activity. Our data support the clinical translation of PTK6 inhibition as a therapeutic strategy for Her2^+^ breast cancers, including those resistant to currently available targeted therapies.

## Additional files

Additional file 1: Figure S1. Lapatinib treatment induces apoptosis of human epithelial growth factor receptor 2 (*Her2*)^+^ breast cancer cells but does not induce Puma. **A** UACC893 and Lapatinib-resistant UACC893R cells were treated with either dimethyl sulfoxide (*DMSO*) or increasing concentrations of Lapatinib (0.5μM−5μM) and counted at the indicated number of days. **B** Lapatinib-sensitive Her2^+^ tumor cells (HCC1954 and SKBR3) were treated with either DMSO or 1 μM Lapatinib for 24 h, stained with propium iodide (PI), and analyzed by fluorescence-activated cell sorting. The percentage of cells in the sub-G1 population is shown. **C** UACC893, UACC893R1, and MDA-MB-453 cells were grown in monolayer cultures, treated with either DMSO or Lapatinib (1 μM) for 24 and 48 h, and lysed. Lysates were probed with the indicated antibodies. **D** SKBR3 and HCC1954 cells were grown in monolayer cultures, treated with either DMSO or Lapatinib (1 μM) for 24 h, and lysed. Lysates were probed with the indicated antibodies. **P* <0.05; ***P* <0.005; *ns* not statistically significant. Additional file 2: Figure S2. PTK6 expression is correlated with genes that negatively regulate apoptosis. Correlation analysis of PTK6 transcript was performed using The Cancer Genome Atlas (TCGA) Breast Cancer Illumina RNAseq V2 level 3 datasets [27]. Gene Ontology (GO) annotation was carried out on genes with expression that correlated with expression of PTK6 at an absolute value for Pearson’s correlation coefficient of 0.3 or greater. The top 20 Gene Ontology Biological Pathway terms are shown, and the overlapping genes in the top GO term. The dotted line represents a nominal *p* value cutoff of 0.05. Additional file 3: Figure S3. PTK6 downregulation induces apoptosis of Lapatinib-resistant human epithelial growth factor receptor 2 (Her2)^+^ breast cancer cells. **A** MDA-MB-453-expressing control or PTK6 shRNA (12) were stained with Annexin-V and propium iodide (PI), and analyzed by fluorescence-activated cell sorting. The percentage of Annexin-V-positive cells is plotted. Statistics were applied to results obtained with triplicate experiments. **B** MDA-MB-453 cells expressing either control or PTK6 shRNAs (C9, 49, 53, and 12) were lysed 96 h after shRNA lentiviral infection. Lysates were probed with antibodies to PTK6 or cleaved PARP. Additional file 4: Figure S4. PTK6 downregulation does not affect the expression of pro- and anti-Bcl2 family members, other than Bim. UACC893R1 and MDA-MB-453 cells expressing either control or two different PTK6 shRNA vectors (C9, 49, or 12) were cultured in the presence of Z-VAD-FMK (50 μM). Cells were lysed and lysates were probed with indicated antibodies. Additional file 5: Figure S5. Bim expression induced by PTK6 downregulation is not secondary to cell death. UACC893R1 cells expressing either control or PTK6 shRNA (49) were treated with Z-VAD-FMK (50 μM) and lysed 72 h after shRNA lentiviral infection. Lysates were probed with antibodies to cleaved PARP or Bim. Additional file 6: Figure S6. Induction of Bim expression is required for PTK6 shRNA-induced apoptosis. UACC893R1 cells expressing either control or two independent Bim shRNA vectors (51 and 54) were superinfected with either control or PTK6 shRNA (49) lentivirus. Cells were lysed at two time points following infection and lysates were probed with antibodies to cleaved PARP or Bim. Additional file 7: Figure S7. Activation of JNK is not required for PTK6 shRNA-induced Bim or apoptosis. **A** UACC893R1 cells expressing either control or PTK6 shRNA were cultured in the presence of Z-VAD-FMK (50 μM). Cells were lysed 48 h after infection and lysates were probed with antibodies to phospho-JNK and phospho-c-Jun. **B** UACC893R1 cells were cultured in the presence of either dimethyl sulfoxide (DMSO) or Anisomycin (as a positive control for c-Jun N-terminal kinase (*JNK*) activation) along with increasing concentrations of SP600125 (JNK inhibitor). Cells were lysed 48 h after DMSO or anisomycin treatment. Lysates were probed with antibodies as indicated. **C** UACC893R1 cells expressing either control or PTK6 shRNA were cultured in the presence of either DMSO or SP600125. Cells were lysed 48 h after PTK6 shRNA lentiviral infection and lysates were probed with antibodies to phospho-c-Jun, cleaved PARP or Bim.

## Abbreviations

3-D: three-dimensional
Bcl2: B cell lymphoma 2
BKS: breast tumor kinase substrate
cleaved PARP: cleaved poly ADP ribose polymerase
DMEM: Dulbecco’s modified Eagle’s medium
DMSO: dimethyl sulfoxide
ECL: enhanced chemiluminescence
EGFR: epidermal growth factor receptor
ER: estrogen receptor
FACS: fluorescence-activated cell sorting
FITC: Fluorescein isothiocyanate
HER2: human epidermal growth factor receptor 2
hsp27: heat shock protein 27
IGF-R1: insulin-like growth factor 1 receptor
IP: immunoprecipitation
JNK: c-Jun N-terminal kinase
MAPK: mitogen-activated protein kinase
PBS: phosphate-buffered saline
PCR: polymerase chain reaction
PI: propidium iodide
PP2A: protein phosphatase-2A
PTK6: protein tyrosine kinase 6
shRNA: short hairpin RNA
TCGA: The Cancer Genome Atlas
TKI: tyrosine kinase inhibitor
β-2M: Beta-2-microglobulin
