# Supplementary figures and images for: PTK6 inhibition promotes apoptosis of Lapatinib-resistant Her2+ breast cancer cells by inducing Bim
Source: Breast Cancer Res. 2015 Jun 19;17(1):86. doi: 10.1186/s13058-015-0594-z (PMC4496943; doi:10.1186/s13058-015-0594-z)

Additional file 1: Figure S1.

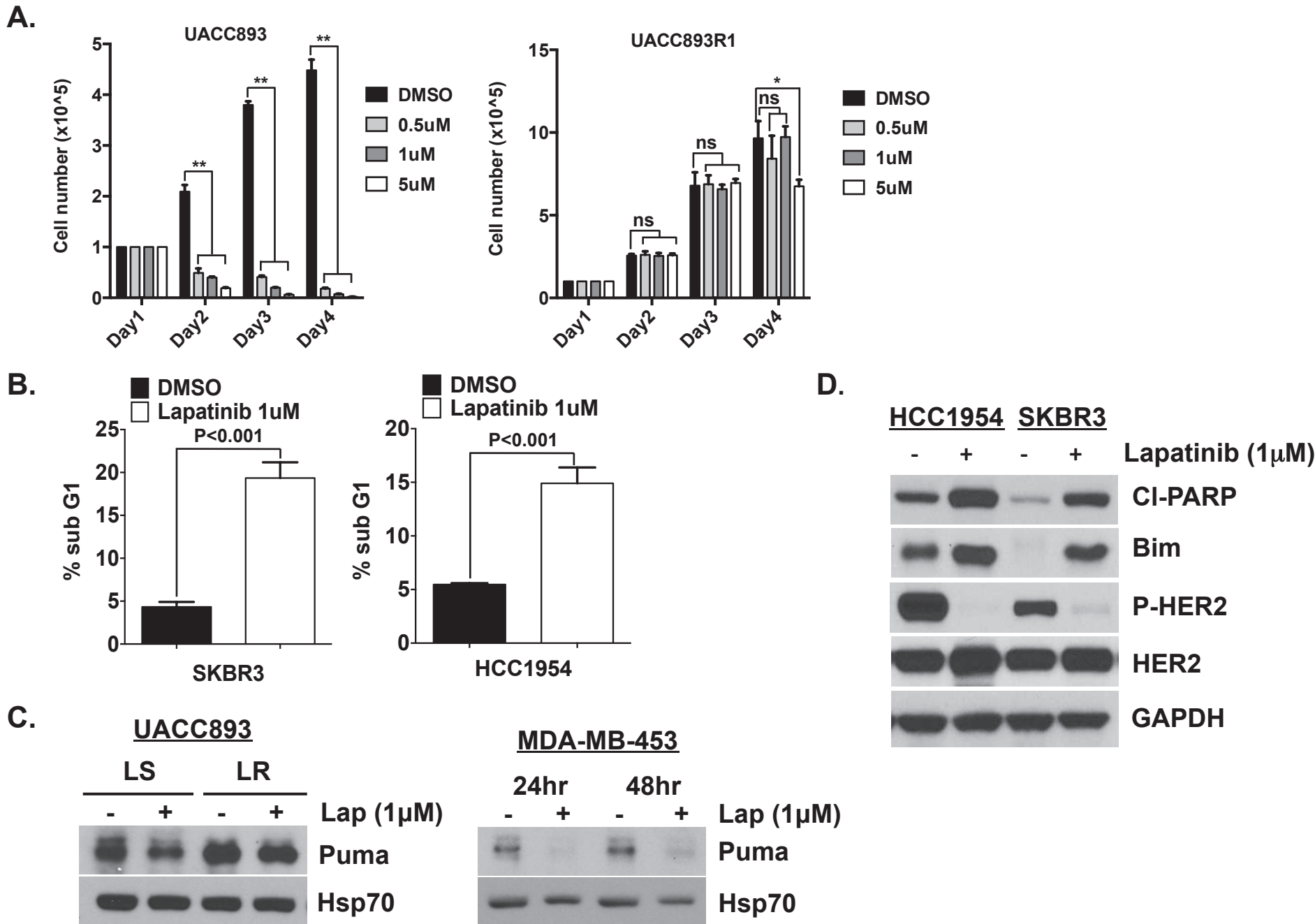

Supplement: Additional file 1: Figure S1. — Lapatinib treatment induces apoptosis of human epithelial growth factor receptor 2 (Her2)+ breast cancer cells but does not induce Puma. A UACC893 and Lapatinib-resistant UACC893R cells were treated with either dimethyl sulfoxide (DMSO) or increasing concentrations of Lapatinib (0.5μM−5μM) and counted at the indicated number of days. B Lapatinib-sensitive Her2+ tumor cells (HCC1954 and SKBR3) were treated with either DMSO or 1 μM Lapatinib for 24 h, stained with propium iodide (PI), and analyzed by fluorescence-activated cell sorting. The percentage of cells in the sub-G1 population is shown. C UACC893, UACC893R1, and MDA-MB-453 cells were grown in monolayer cultures, treated with either DMSO or Lapatinib (1 μM) for 24 and 48 h, and lysed. Lysates were probed with the indicated antibodies. D SKBR3 and HCC1954 cells were grown in monolayer cultures, treated with either DMSO or Lapatinib (1 μM) for 24 h, and lysed. Lysates were probed with the indicated antibodies. *P <0.05; **P <0.005; ns not statistically significant. [file 13058_2015_594_MOESM1_ESM.pdf]

## Additional file 2: Figure S2.

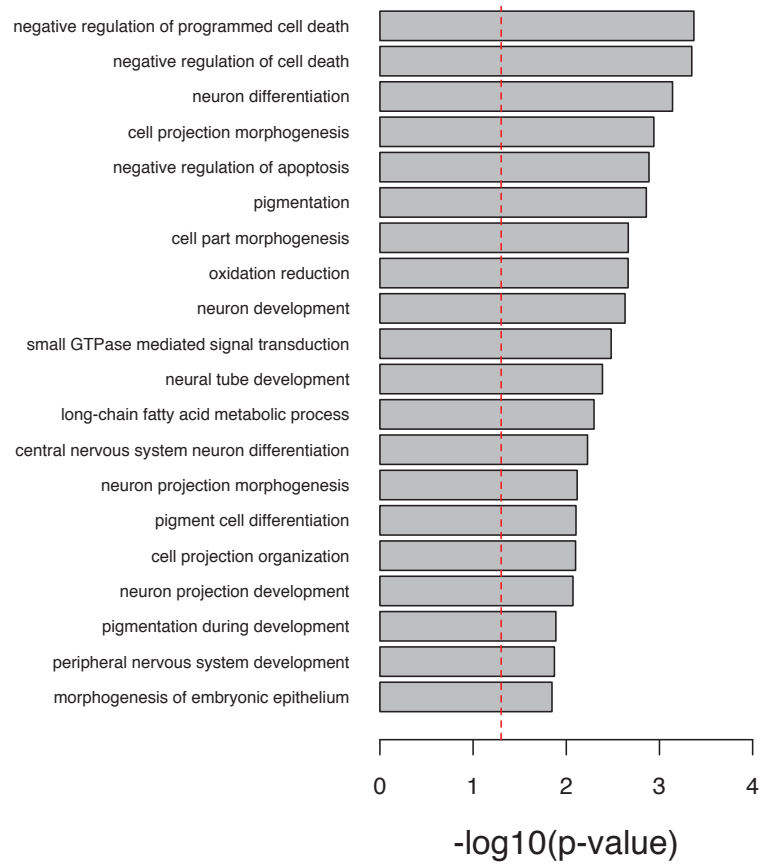

Supplement: Additional file 2: Figure S2. — PTK6 expression is correlated with genes that negatively regulate apoptosis. Correlation analysis of PTK6 transcript was performed using The Cancer Genome Atlas (TCGA) Breast Cancer Illumina RNAseq V2 level 3 datasets [27]. Gene Ontology (GO) annotation was carried out on genes with expression that correlated with expression of PTK6 at an absolute value for Pearson’s correlation coefficient of 0.3 or greater. The top 20 Gene Ontology Biological Pathway terms are shown, and the overlapping genes in the top GO term. The dotted line represents a nominal p value cutoff of 0.05. [file 13058_2015_594_MOESM2_ESM.pdf]

### Additional file 3: Figure S3

**A.**

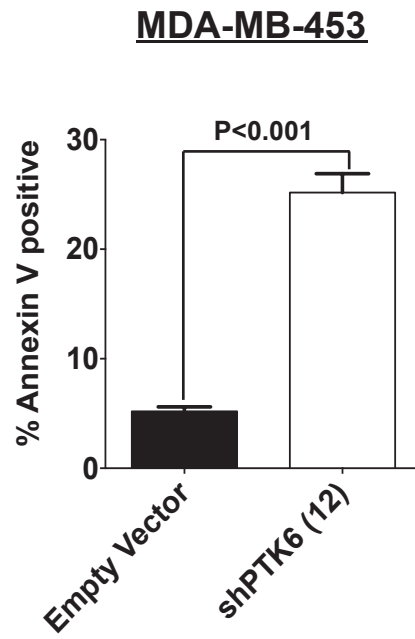

**B.**

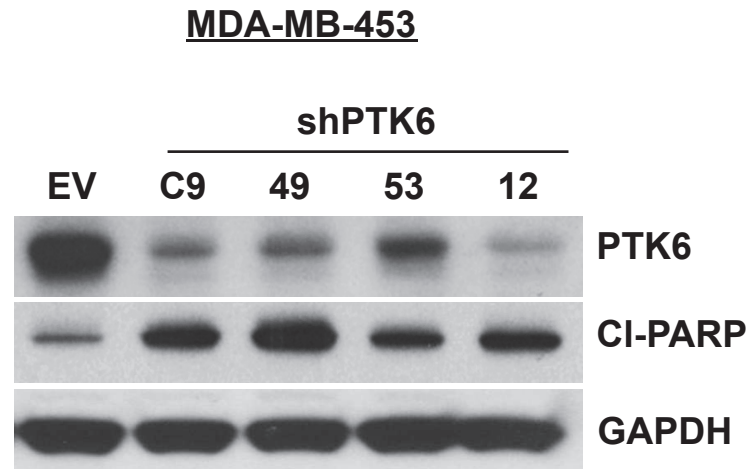

Supplement: Additional file 3: Figure S3. — PTK6 downregulation induces apoptosis of Lapatinib-resistant human epithelial growth factor receptor 2 (Her2)+ breast cancer cells. A MDA-MB-453-expressing control or PTK6 shRNA (12) were stained with Annexin-V and propium iodide (PI), and analyzed by fluorescence-activated cell sorting. The percentage of Annexin-V-positive cells is plotted. Statistics were applied to results obtained with triplicate experiments. B MDA-MB-453 cells expressing either control or PTK6 shRNAs (C9, 49, 53, and 12) were lysed 96 h after shRNA lentiviral infection. Lysates were probed with antibodies to PTK6 or cleaved PARP. [file 13058_2015_594_MOESM3_ESM.pdf]

Additional file 4: Figure S4.

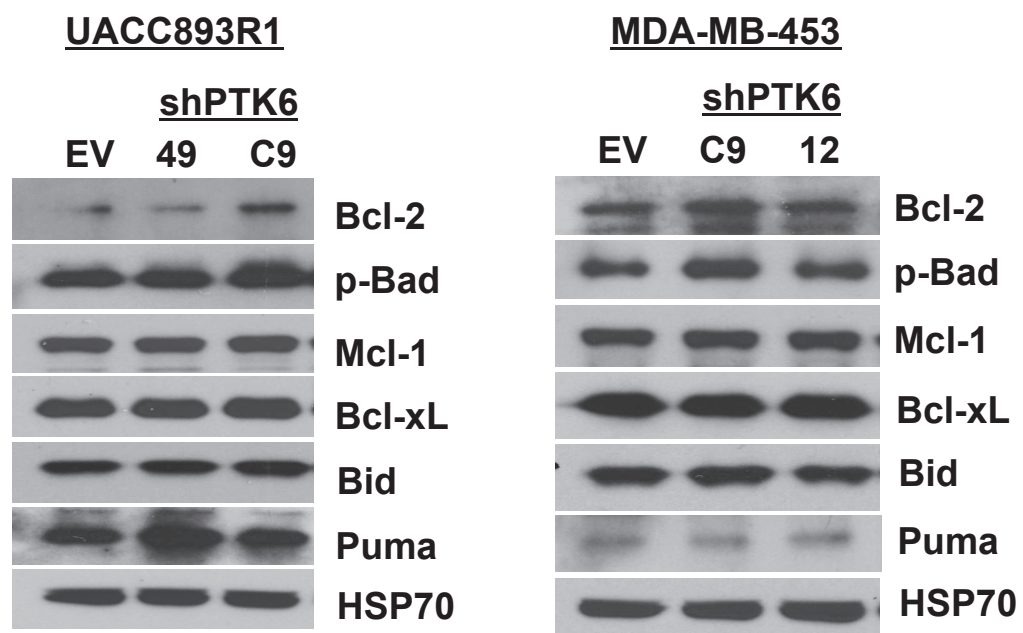

Supplement: Additional file 4: Figure S4. — PTK6 downregulation does not affect the expression of pro- and anti-Bcl2 family members, other than Bim. UACC893R1 and MDA-MB-453 cells expressing either control or two different PTK6 shRNA vectors (C9, 49, or 12) were cultured in the presence of Z-VAD-FMK (50 μM). Cells were lysed and lysates were probed with indicated antibodies. [file 13058_2015_594_MOESM4_ESM.pdf]

**Additional file 5: Figure S5.**

**UACC893R1**

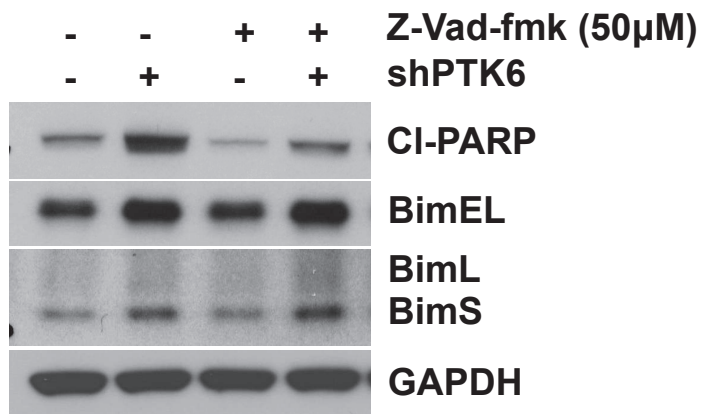

Supplement: Additional file 5: Figure S5. — Bim expression induced by PTK6 downregulation is not secondary to cell death. UACC893R1 cells expressing either control or PTK6 shRNA (49) were treated with Z-VAD-FMK (50 μM) and lysed 72 h after shRNA lentiviral infection. Lysates were probed with antibodies to cleaved PARP or Bim. [file 13058_2015_594_MOESM5_ESM.pdf]

**Additional file 6: Figure S6.**

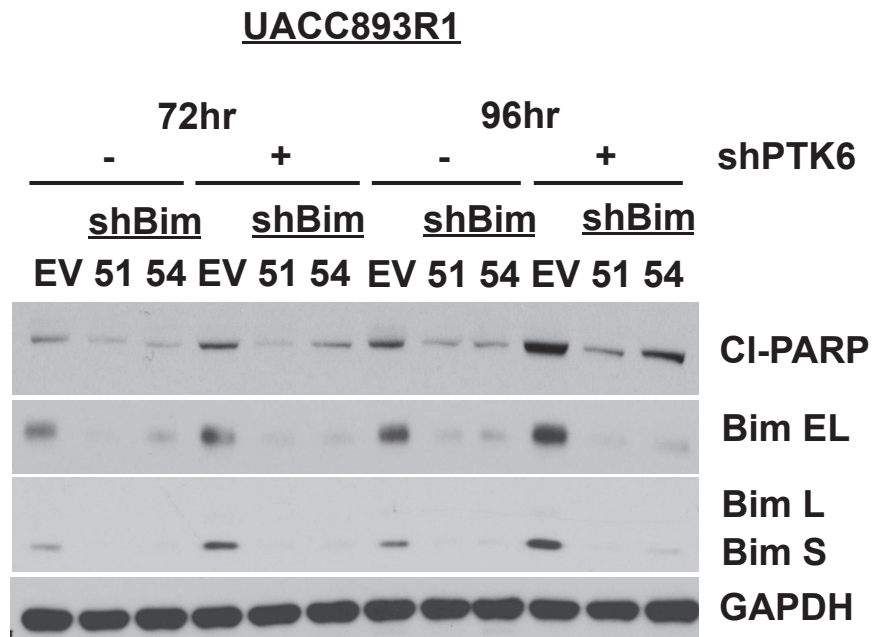

Supplement: Additional file 6: Figure S6. — Induction of Bim expression is required for PTK6 shRNA-induced apoptosis. UACC893R1 cells expressing either control or two independent Bim shRNA vectors (51 and 54) were superinfected with either control or PTK6 shRNA (49) lentivirus. Cells were lysed at two time points following infection and lysates were probed with antibodies to cleaved PARP or Bim. [file 13058_2015_594_MOESM6_ESM.pdf]

Additional file 7: Figure S7.

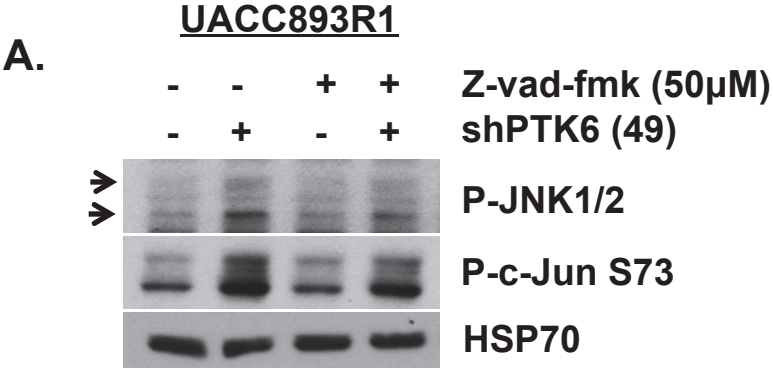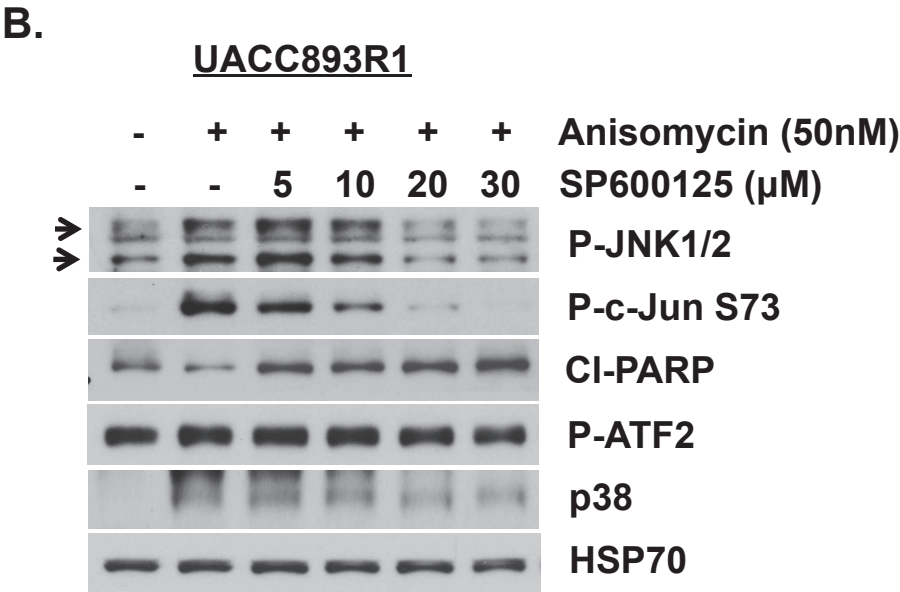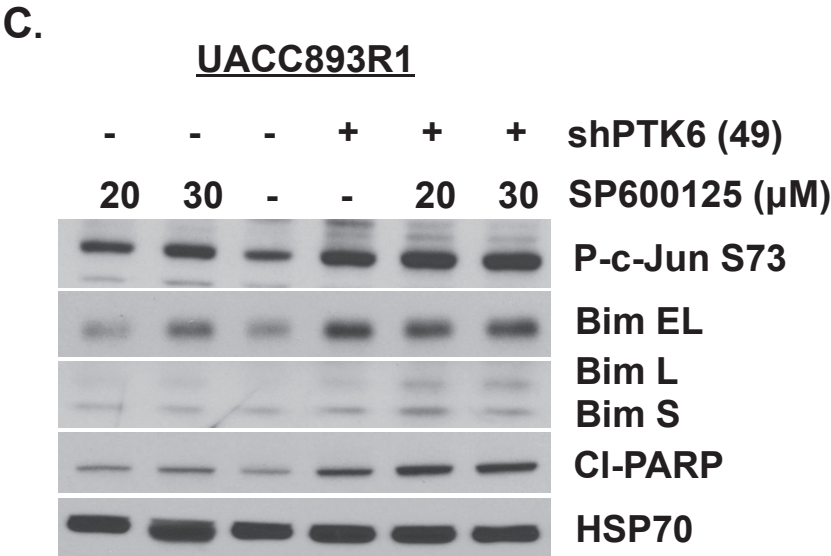

Supplement: Additional file 7: Figure S7. — Activation of JNK is not required for PTK6 shRNA-induced Bim or apoptosis. A UACC893R1 cells expressing either control or PTK6 shRNA were cultured in the presence of Z-VAD-FMK (50 μM). Cells were lysed 48 h after infection and lysates were probed with antibodies to phospho-JNK and phospho-c-Jun. B UACC893R1 cells were cultured in the presence of either dimethyl sulfoxide (DMSO) or Anisomycin (as a positive control for c-Jun N-terminal kinase (JNK) activation) along with increasing concentrations of SP600125 (JNK inhibitor). Cells were lysed 48 h after DMSO or anisomycin treatment. Lysates were probed with antibodies as indicated. C UACC893R1 cells expressing either control or PTK6 shRNA were cultured in the presence of either DMSO or SP600125. Cells were lysed 48 h after PTK6 shRNA lentiviral infection and lysates were probed with antibodies to phospho-c-Jun, cleaved PARP or Bim. [file 13058_2015_594_MOESM7_ESM.pdf]
